# Therapeutic Endoscopic Ultrasonography: Intratumoral Injection for Pancreatic Adenocarcinoma

**DOI:** 10.1155/2013/207129

**Published:** 2013-03-27

**Authors:** Lawrence A. Shirley, Laura K. Aguilar, Estuardo Aguilar-Cordova, Mark Bloomston, Jon P. Walker

**Affiliations:** ^1^Division of Surgical Oncology, The Ohio State University Wexner Medical Center, Columbus, OH 43210, USA; ^2^Advantagene, Inc., Auburndale, MA 02466, USA; ^3^Division of Gastroenterology, Hepatology, and Nutrition, The Ohio State University Wexner Medical Center, Columbus, OH 43210-1267, USA

## Abstract

Pancreatic adenocarcinoma is an aggressive disease that has poor outcomes despite maximal traditional therapies. Thus, treatment of this cancer demands innovative strategies to be used in addition to standing therapies in order to provide new avenues of care. Here, we describe the technique of using endoscopic ultrasound in order to directly inject both novel and conventional therapies into pancreatic tumors. We detail the rationale behind this strategy and the many benefits it provides. We then describe our technique in detail, including our experience injecting the AdV-tk adenoviral vector to create an in situ vaccine effect.

## 1. Rationale

Pancreatic adenocarcinoma is the 11th most common cause of cancer, but the 5th leading cause of cancer deaths [1]. The vast majority of patients present with cancer untreatable by surgical excision owing to locally advanced or metastatic disease; even those who are able to undergo resection almost always succumb to this disease with 94% dying within 5 years [1]. Thus, novel treatment methods are desperately needed in order to improve outcomes. Here, we present one such technique, the injection of AdV-tk into the pancreatic tumor using endoscopic ultrasonography (EUS) prior to resection. AdV-tk is a replication defective adenoviral vector expressing the Herpes simplex virus thymidine kinase gene that renders tumor cells susceptible to the cytotoxic effects of antiherpetic prodrugs, such as valacyclovir, and more importantly induces a potent local and systemic antitumor immune response [2]. 

 Besides the novelty of this therapy, EUS injection of pancreatic tumors provides an advantage over other treatment modalities in that it can overcome the anatomical constraints inherent to gaining access to the pancreatic parenchyma. Located in the retroperitoneum and adjacent to large mesenteric vessels, the pancreas is not easily accessed by other minimally invasive techniques such as computed tomography (CT) guided injection. Other techniques, such as laparoscopic or open surgical techniques, are much more invasive, bringing with them potential morbidity and longer hospital stays. An additional theoretical advantage of direct injection over intravenous or oral administration of medications is that pancreatic adenocarcinoma is relatively hypovascular and creates an extensive desmoplastic reaction [3], thus impeding the access of traditional chemotherapy to tumor cells. Tissue-specific targeting of agents, such as viral vectors, can be accomplished easily by direct tumoral injection without having to overcome pancreatic tumor hypovascularization or evade systemic antiviral immunity to get the vector to its target tissue.

Several recent studies have shown initial success with injection of various medications and therapies into pancreatic tumors using this technique. Such examples include the allogenic mixed lymphocyte culture cytoimplant [4], the replication-selective adenovirus ONYX-015 [5], an adenovector encoding tumor necrosis factor TNFerade [6], the traditional systemic chemotherapeutic agent gemicitabine [7], and tumor antigen loaded dendritic cells [8]. All have shown some initial degree of success, showing this technique to be of practical therapeutic value. Herein we describe our institution's method of performing intratumoral injection into pancreatic tumors.

## 2. Procedural Details

As part of a phase I clinical trial (NCT00638612), a replication-defective adenoviral vector (AdV-tk, Advantagene, Inc, Auburndale, MA) was injected prior to surgery for resectable (Arm A) or prior to and during chemoradiation for locally advanced (Arm B) pancreatic cancer. The results of this trial will be reported separately. After cytologic confirmation of pancreatic cancer, tumors were injected with AdV-tk under EUS guidance.

The process of intratumoral injections under the guidance of EUS, when performed by an experienced endosonographer, is fairly straightforward. The required equipment includes a curvilinear echoendoscope, an endoscopic ultrasound processor with Doppler technology, and a 22-gauge fine needle aspiration (FNA) needle. The procedure is performed in the endoscopy unit and typically does not require fluoroscopy. If initial sampling is not required, the procedure time is approximately 15–30 minutes. 

Most patients tolerate standard conscious sedation utilizing a narcotic, such as fentanyl or meperidine, and a benzodiazepine, typically midazolam. However, in our experience, patients who are young, have a history of chronic narcotic or alcohol use, or have previous anxiolytics usage require higher levels of sedation to maintain comfort during the procedure. Thus, monitored anesthesia care or general anesthesia may also be required. Especially in cases of conscious sedation, a topical anesthetic is often utilized, as the intubation of the esophagus with the larger caliber echoendoscope can be uncomfortable. Since precise needle placement is required to insure good distribution of vector in the tumor, adequate anesthesia is crucial. 

For initial identification and characterization of the pancreatic mass lesion, either a radial or curvilinear echoendoscope can be utilized. Both echoendoscopes can be used for identification, characterization, measurements, and evaluation for surrounding vasculature. However, these are the limitations of the radial echoendoscope. Sampling and fine-needle injection requires the use of the curvilinear echoendoscope. 

EUS guided fine needle injection is performed in a similar fashion to standard EUS with FNA. The esophagus is carefully intubated and the echoendoscope is passed into the stomach and duodenum where the pancreatic examination occurs. Echoendoscope position in the stomach allows for visualization of the pancreas genu, body, and tail. It is from this location that the celiac axis can be evaluated for lymphadenopathy and the left liver lobe can be evaluated for evidence of metastatic lesions. Echoendoscope position in the duodenum bulb allows for visualization of the pancreas head and genu, as well as the extrahepatic biliary tree. Finally, echoendoscope position in the second portion in the duodenum allows for visualization of the pancreas head, uncinate process, and distal common bile duct, as well as endoscopic visualization of the major and minor papilla.

**Figure 1:**
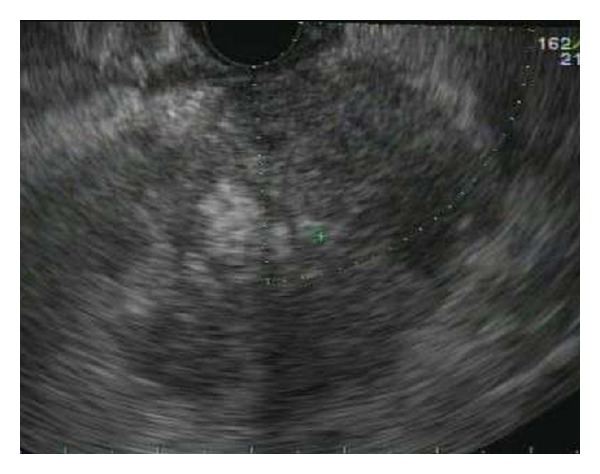
Endoscopic ultrasound image of a tumor in the head of the pancreas prior to intratumoral injection.

**Figure 2:**
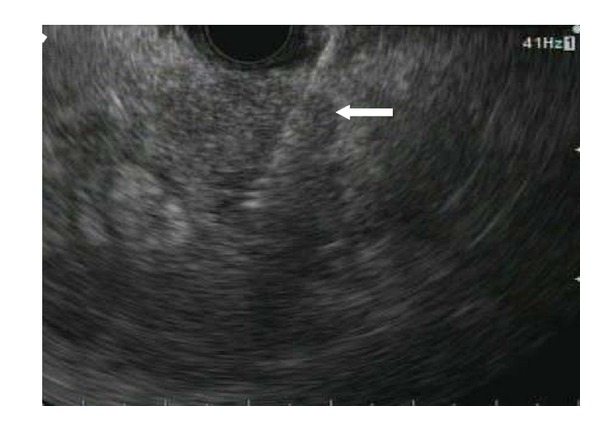
The same pancreatic tumor from Figure 1 being injected with intratumoral therapy. The white arrow indicates the hyperechoic shadow of the fine needle entering the tumor.

Once the mass has been identified (Figure 1), it is important to obtain an accurate measurement of the largest plane. In our trial, this measurement was utilized to estimate tumor volume in order to calculate volume of AdV-tk for injection. Based on previous preclinical data and treatment with previous tumors, we chose to inject approximately 20–40% of the total tumor volume. AdV-tk was administered by dividing the tumor into up to 4 quadrants and then injecting 1 mL into each quadrant. For tumors with estimated volumes greater than 20 cm^3^, an accessible region where the sum of the major and minor axis was approximately 6 cm was chosen and this area was divided into 4 quadrants for injection. Use of the installed Doppler technology also assures there are no arterial or venous structures that may interfere with the needle passage into the tumor. If such interference is present, the scope may need to be repositioned to obtain a similar view from another angle or the procedure may need to be aborted. Once the tumor size and subsequent injection volume has been calculated, the appropriate volume is drawn into a syringe. The 22-gauge FNA needle is primed with approximately 0.5 cc of injection solution and passed into the working channel of the echoendoscope. Under ultrasound guidance, the needle is passed into the tumor (Figure 2). In order to distribute vector throughout the tumor, a total of four passes were used in a fanning technique along the single largest plane of the tumor with 1/4 volume injected at each pass. The needle tip was first passed into the back of the tumor. As the needle was slowly withdrawn, 1/4 of the solution is injected into the tunnel created by the needle. Patients were monitored in the recovery suite as per standard protocol and then discharged to home.

The EUS and needle passage procedures tend to be very safe with a less than 1% risk of immediate and delayed complication. These include perforation, bleeding, and aspiration, in addition to standard risk associated with sedations, such as hypoxia, arrhythmia, and hypotension. The risk of bleeding is minimal due to the use of Doppler technology, as well as the small 22G needle caliber, and our experience has yet to produce bleeding serious enough to require transfusion. The risk of infectious complications related to needle passage is minimal, and prophylactic antibiotics are not typically recommended. Risks related to the injection solution ultimately depend on the solution or chemotherapeutic agent and are usually related to inflammatory complication, such as pancreatitis. No procedure-related complications occurred in this trial with AdV-tk (to be reported separately).

## 3. Conclusions

Pancreatic cancer is a highly lethal form of cancer, and standard therapies provide minimal long-term benefit. Thus, novel strategies are vitally important in order to improve outcomes. Here we have described intratumoral injection of pancreatic masses via endoscopic techniques. This technique provides the ability to treat the pancreas in a direct and relatively minimally invasive manner, with a very low incidence of procedural-related complications. It bypasses the poor parenchymal penetration of systemic chemotherapy and the morbidities inherent to laparoscopic or open surgical procedures, as well as giving the ability to administer therapies that would be prohibitive to give systemically. Giving innovative treatments in such a specific and novel manner could provide hope in treating this otherwise overwhelming disease.
